# Myriad Pathways to Universality: How Widely Sourced Data, Use of Frameworks and Innovative Analytic Methods Help Tackle Immunization Inequality

**DOI:** 10.3390/vaccines14010083

**Published:** 2026-01-13

**Authors:** Devaki Nambiar, Ahmad Reza Hosseinpoor, Nicole Bergen, M. Carolina Danovaro-Holliday, Ibrahim Dadari, Hope L. Johnson

**Affiliations:** 1 Department of Data, Digital Health, Analytics and AI, World Health Organization, 1211 Geneva, Switzerland; hosseinpoora@who.int (A.R.H.); bergenn@who.int (N.B.); 2 Department of Immunization, Vaccines and Biologicals (IVB), World Health Organization, 1211 Geneva, Switzerland; danovaroc@who.int; 3 United Nations Children’s Fund (UNICEF), New York, NY 10017, USA; idadari@unicef.org; 4 Measurement, Evaluation and Learning Department, Gavi, the Vaccine Alliance, 1218 Geneva, Switzerland; hjohnson@gavi.org

Great strides have been made in the area of immunization over the past several decades. Childhood vaccination averted 154 million deaths, including 146 million among children younger than 5 years between 1974 and 2024 [1]. These milestones are particularly important as countries seek to return to pre-COVID-19 immunization levels. A joint release by the World Health Organization (WHO) and the United Nations Children’s Fund (UNICEF) in July 2025 highlighted some ongoing challenges, including inequalities in access to vaccines, funding gaps, challenges in conflict/fragile settings, misinformation, and the need to strengthen data and surveillance systems [2]. Furthermore, the mid-term review of the Immunization Agenda 2030 (IA2030), which is a global strategy to not leave anyone behind with vaccines, also highlighted how the world is off-track to reach the objectives set in IA2030 and called for intensified efforts to increase coverage and equity [3]. This 2025 Special Issue on Inequality in Immunization is the third iteration of an effort to surface and deepen the exploration of immunization inequalities across global contexts, using myriad methodologies. It also seeks to showcase insights into the design of equity-oriented interventions to universalize immunization. This Special Issue comprises 15 papers including 3 reviews, 10 research articles and 2 perspective pieces.

## Geographical Representation

The papers in this Special Issue provide insight into and evidence for immunization inequalities across diverse subnational, national and cross-country contexts. We have strong representation from author teams working at the country level, including in the Democratic Republic of Congo (DRC) (Nimpa et al.), India (Lavalekar et al.), Jamaica (Webster-Kerr et al.), Madagascar (Demare et al.), Nigeria (Attahiru et al., Gordon et al., Umar et al.) and Zambia (Powell et al.). Phillips et al.’s decomposition analysis drew from 295 Demographic and Health Surveys (DHS) from 80 low- and middle-income countries (LMICs) in their exploration of childhood immunization data. Likewise, DHS datasets were used in a study focused on maternal tetanus across 39 countries in Kirkby et al.’s analysis. Mansilla and colleagues’ gender analysis spans 29 UNICEF focus countries, and Oyugi et al. drew upon 36 implementation research projects in 13 LMICs, while Mendez-Lopez and colleagues explore economic inequalities in full immunization within 10 countries of the Western Pacific region. Another study authored by Githaiga and colleagues focuses on human papillomavirus (HPV) vaccination services among involuntary migrants from 35 countries who have migrated to Australia, Canada, Denmark, Greece, Lebanon, Nepal, the Netherlands, Türkiye and the United States of America. Bergen et al. reviewed global reports, publications and trainings on the state of inequality in childhood immunization that drew on various sets of data.

## Indicators of Focus and Dimensions of Inequality

Much of the focus of the contributions to this Special Issue is on childhood vaccination, either looking at the receipt of multiple vaccinations as an outcome, or focusing on those left behind using indicators of under-vaccination, such as zero dose. Phillips et al. explored the relationship between indicators of zero dose (no diphtheria–tetanus–pertussis (DTP) doses received), missed (at least one dose of Bacille Calmette–Guérin (BCG), polio or measles-containing vaccine (MCV) but no doses of DTP), and drop out (at least 1 but not all 3 doses of DTP) of DTP3 immunization, remarking on key differences between the “left behind” groups they represent. Other papers examined inequalities in the context of specific immunization programmes or vaccines, such as pneumococcal vaccines (Nimpa et al.) or maternal tetanus immunization (Kirkby et al.). Githaiga and colleagues’ paper on HPV vaccination among involuntary migrants is a novel analysis that specifically points out the lack of attention paid to adolescent immunization, particularly in this underserved population subgroup. Papers from India and Jamaica highlight COVID-19 vaccination gaps and survival gains, respectively (Lavalekar et al., Webster-Kerr et al.). Two review papers authored by UNICEF and WHO colleagues, respectively, lay out the landscape of inequality analysis in the analytical and programmatic work of these two agencies, which have placed great emphasis on advancing immunization equity (Mansilla et al., Bergen et al.).

The articles collectively explore a range of dimensions of inequality, reflecting broader calls to consider diverse forms of inequality in immunization. Geographical inequality was covered by several studies, exploring how immunization indicators differed between urban and rural areas, or on the basis of administrative units such as regions, wards, districts and health facility catchment zones (Demare et al., Powell et al., Attahiru et al., Gordon et al., Webster-Kerr et al.). Two studies provided detailed analyses of gender inequalities in immunization—Kirkby et al. exploring the components of the SWPER index and Mansilla et al. looking at gender-related barriers and drivers. While Mendez-Lopez and colleagues maintain a dedicated focus on economic-related inequalities, Kirkby et al. and Gordon et al. consider this dimension alongside other forms of inequality. Age, education level or ethnicity is reflected in a number of the analyses (Attahiru et al., Gordon et al., Webster-Kerr et al.). In a few instances, research articles add to emerging bodies of evidence surrounding less-commonly explored drivers of inequality such as disability in Lavalekar and colleagues’ work, and migrant populations, as in Githaiga et al.’s paper.

## Methods, Frameworks and Key Findings

This Special Issue features both papers focused on identifying inequalities and those exploring equity-oriented interventions in immunization. Eight papers characterize the nature of inequalities (Kirkby et al., Mendez-Lopez et al., Gordon et al., Umar et al., Mansilla et al., Lavalekar et al., Phillips et al., and Githaiga et al.), while seven papers explore equity-enhancing interventions and approaches (Nimpa et al., Attahiru et al., Powell et al., Demare et al., Webster-Kerr et al., Oyugi et al., and Bergen et al.). Across the Special Issue, contributions make use of innovative methodological approaches and apply frameworks in novel ways, advancing scholarship in this area.

Kirkby et al. captured a wide range of multidimensional gender barriers in their study of maternal tetanus coverage inequalities. Higher levels of women’s empowerment as well as greater decision-making autonomy, as measured by the Survey-based Women’s emPowERment (SWPER) index, were associated with higher odds of maternal tetanus coverage. Wealth-related inequality in immunization was also seen, with greater household wealth associated with greater odds of maternal tetanus vaccine uptake. Indeed, wealth was also explored in a study by Mendez-Lopez, who used the summary measures of inequality Population Attributable Risk (PAR) and Population Attributable Fraction (PAF) to estimate childhood immunization inequality in ten countries from the Western Pacific region. In five countries (namely Fiji, Lao PDR, Papua New Guinea, Samoa and Tonga), national coverage increased as wealth-based inequalities were minimized over time (Mendez-Lopez et al.).

UNICEF’s Global Gender Analysis Tool was used by Mansilla et al. to review barriers to gender equity in immunization access, describing barriers at the system, service, community and household levels. Unique challenges emerged in fragile and conflict-affected geographies. This is the first study to synthesize gender barriers to immunization using data generated from UNICEF-supported gender analyses, which were conducted across multiple income levels and geographies.

Gordon employed the Levesque Framework for Healthcare Access to explore how maternal exposure to media might be associated with healthcare-seeking behaviours in Nigeria. The authors report a significant association between region and immunization status in Nigeria, and also an association between television exposure and higher completed childhood immunization (although model fit estimates suggested that only a fifth of the variance was explained by the model developed).

Meanwhile, studies led by Attahiru and Lavalekar employed the WHO Behavioural and Social Drivers for Immunization (BeSD) framework [4]. In India, BeSD helped identify a range of barriers to vaccination access among transgender and persons living with disability including issues of invisibility in health systems, a lack of trust, and mobility and accessibility constraints. In Nigeria, BeSD was employed alongside Lot Quality Assurance Sampling (LQAS) to identify barriers to delayed vaccination (including low caregiver education, rurality and negative vaccine perceptions). It is noteworthy that a lack of trust in health systems and providers emerged as a hindrance to immunization equity across multiple vaccine types.

Powell et al. carried out a GIS-based study in five schools within a single district of Zambia using, for the first time in this setting, school entry vaccination checks (which is a scalable way of identifying under-vaccinated children). Their findings of variations in under-vaccination across school catchment areas suggested a need for targeted strategies in certain areas, and a more universal approach in others.

Focusing on zero dose reduction at the local administrative level in Southern Madagascar, Demare and colleagues used the RE-AIM and PRISM implementation science frameworks to create an evaluation framework that supports local adaptation in low-coverage settings. Drawing from quantitative programme monitoring and qualitative community insights, it proposes eight design principles for immunization evaluation efforts, which are particularly relevant in fragile settings: use disaggregated data; embed equity into frameworks; pair numbers with narratives; design for adaptation; centre local perspectives; define and measure marginalization; plan for equity before measuring impact; and include feedback loops.

Interested in the population-level impact of universalizing the 13-valent pneumococcal vaccination (PCV13), Nimpa and colleagues developed a model drawing on a systematic review and the Lives Saved Tool (LiST). The study found that PCV13 vaccination coverage in DR Congo was 79.0% in 2022, at which point 113,359 new cases of severe pneumonia and 17,255 deaths attributable to severe pneumonia were prevented in the country.

In a novel application of decomposition analysis, Phillips and colleagues have noted that, for about 2.4 million children across 80 countries, coverage variation is due to zero dose in some years and locations and to missed dose in others. This finding suggests that differential strategies should be applied where zero dose is a greater contributor as well as where missed doses are a challenge. In Jamaica, Webster-Kerr and colleagues concluded that their survival analysis is suggestive of greater COVID-19 vaccine effectiveness among younger, female and more recently immunized persons, which again is a novel methodological application allowing for the identification of who is left behind regarding the benefit of COVID-19 vaccination, inviting further exploration of clinical, health system and other drivers of this inequality.

## Key Emerging Themes

The highly diverse collection of papers in this Special Issue points to some key insights at this inflection point in the global march towards universal immunization. First, having and analyzing disaggregated data remains critically important across various types of datasets and methodological approaches, to understand and address inequality. As Bergen and colleagues point out, there are looming threats to the sustained availability of disaggregated data (i.e., from sources like DHS, a programme that at the time of this writing is only partially operational [5]), and even as we develop creative ways to synthesize and use programme data, it remains important to ensure that robust disaggregated data continue to be available for monitoring and tackling inequalities.

Taken together, findings related to a variety of outcomes help shed light on immunization inequality: coverage of a single index vaccination, full immunization, under-vaccination and zero dose. These indicators must continue to be standardized and their relationships with health outcomes and effective coverage tested—in this way, we may ascertain which indicators allow the effective development of programmatic and policy responses. Across papers, we found that economic status and geography are prominent dimensions of inequality, especially at the subnational level, including for fragile- and conflict-affected, and migrating populations. Understanding these intersections, many of which are gendered, will be particularly important for guiding policy and planning activities. Moreover, hyperlocal and place-based areas (e.g., subnational units and schools) appear to be highly appropriate for site interventions. This is a logical response in light of the geographic inequalities emerging across studies as well as the variation in how countries have attempted to bridge gaps in coverage. The localization of data analysis and intervention is also mentioned in the mid-term IA2030 review [3].

This Special Issue highlights a range of methodological approaches, conceptual frameworks and tools that may be useful in surfacing inequities and helping to address them. The compelling approaches and findings from these studies suggest that much more innovation is possible in carrying out inequality analyses, and particularly in informing the “solution” space in broadening immunization coverage in an equitable manner.

Global policy frameworks for immunization and strong advocacy in the past quarter century have created an environment well-suited to continuous academic and implementation-oriented analysis of immunization inequalities. As the IA2030 and Sustainable Development Goals milestones approach, we are faced with a changed geopolitical context and slowing progress in addressing inequities. Our Special Issue suggests that there are areas where we must stand firm and areas where we must keep moving. A firm and persistent focus on disaggregated data, and category or subnational targeting is central to foregrounding equity in immunization going forward. There can be no inequality analysis without it. Apart from this non-negotiable, this Special Issue suggests that we may be more creative and ambitious in how we define the scope, indicators and analytic approaches for inequality analysis in immunization, using myriad, fit-for-purpose approaches to inform fit-for-purpose solutions. There are, indeed, myriad paths to universality.
